# Supplementary material for: Mutant IDH impairs chromatin binding by PDGFB to promote chromosome instability
Source: bioRxiv. 2025 Feb 25:2025.02.20.639365. Preprint. [Version 1] doi: 10.1101/2025.02.20.639365 (PMC11888161; doi:10.1101/2025.02.20.639365)
Supplement: Supplement 1 [file NIHPP2025.02.20.639365v1-supplement-1.pdf]

bioRxiv preprint doi: https://doi.org/10.1101/2025.02.20.639365; this version posted February 25, 2025. The copyright holder for this preprint (which was not certified by peer review) is the author/funder, who has granted bioRxiv a license to display the preprint in perpetuity. It is made available under aCC-BY 4.0 International license.

Supplemental Figures and Tables

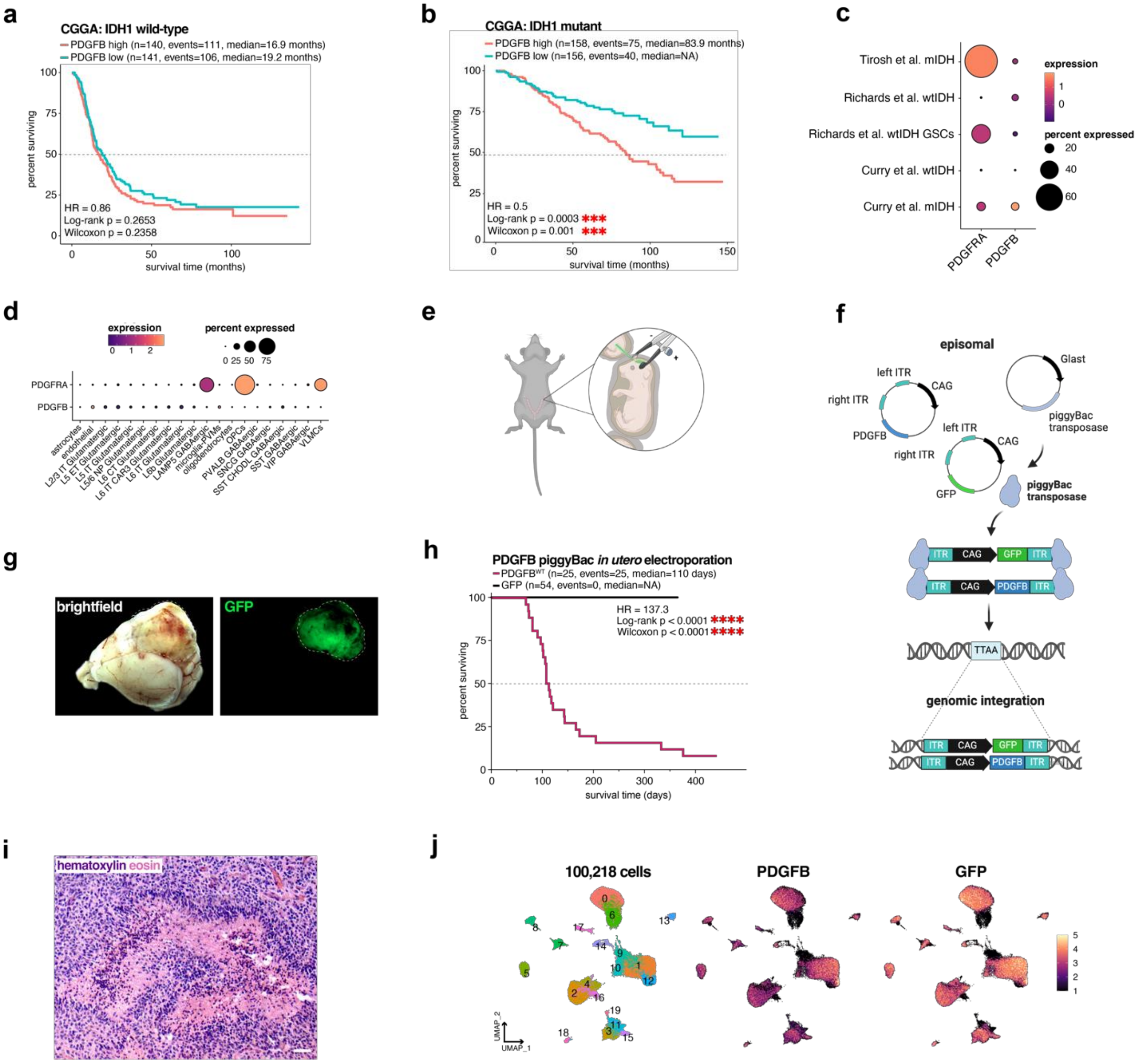

bioRxiv preprint doi: https://doi.org/10.1101/2025.02.20.639365; this version posted February 25, 2025. The copyright holder for this preprint (which was not certified by peer review) is the author/funder, who has granted bioRxiv a license to display the preprint in perpetuity. It is made available under aCC-BY 4.0 International license.

Figure S1. PDGFB PDGFB drives mouse gliomagenesis in the absence of mutations. (a) Kaplan-Meier survival analysis of wtIDH glioma patients from the CGGA split on median *PDGFB* expression. No significant difference in survival outcome is observed between high (n=140; events=111; median=16.9 months) and low (n=141; events=106; median=19.2 months) *PDGFB* expression groups (HR=0.86; log-rank $p<0.2653$; Wilcoxon $p<0.2358$). (b) Kaplan-Meier survival analysis of mtIDH glioma patients from the CGGA split on median *PDGFB* expression. High *PDGFB* expression (n=158; events=75; median=83.9 months) is associated with worse survival outcomes when compared to low *PDGFB* expression (n=158; events=40; median=NA) (HR=0.5; log-rank ***$p<0.0003$; Wilcoxon ***$p<0.001$). (c) Dot plot of scRNA-seq data from previously published datasets showing *PDGFRA* and *PDGFB* expression are higher in mIDH glioma as compared to wtIDH glioma. (d) Dot plot of non-tumor human brain scRNA-seq data from the Allen Brain Atlas shows *PDGFB* is minimally expressed in the non-tumor human brain and *PDGFRA* is enriched in OPCs. (e) Schematic representation of the intraventricular injection used in the piggyBac *in utero* electroporation system. (f) Schematic representation of the expression vectors used in the piggyBac *in utero* electroporation system. (g) Brightfield and fluorescent images of whole brains showing end-stage tumors in PDGFB^{wt} mice; white dashed line denotes tumor. (h) Kaplan-Meier survival analysis of PDGFB^{wt} (n=25; events=25; median=110 days) and GFP alone (n=54; events=0; median=NA) mice (HR=137.3; log-rank ****$p<0.0001$; Wilcoxon ****$p<0.0001$). (i) H&E staining of an end-stage PDGFB^{wt} (P100) tumors shows histopathology consistent with high-grade glioma; scale bar = 50 microns. (j) UMAPs of scRNA-seq (n=100,218 cells; n=3 mice) data shows GFP and PDGFB overexpression transcripts co-localize to the same cells. CGGA: Chinese Glioma Genome Atlas; H&E: hematoxylin and eosin.

bioRxiv preprint doi: https://doi.org/10.1101/2025.02.20.639365; this version posted February 25, 2025. The copyright holder for this preprint (which was not certified by peer review) is the author/funder, who has granted bioRxiv a license to display the preprint in perpetuity. It is made available under aCC-BY 4.0 International license.

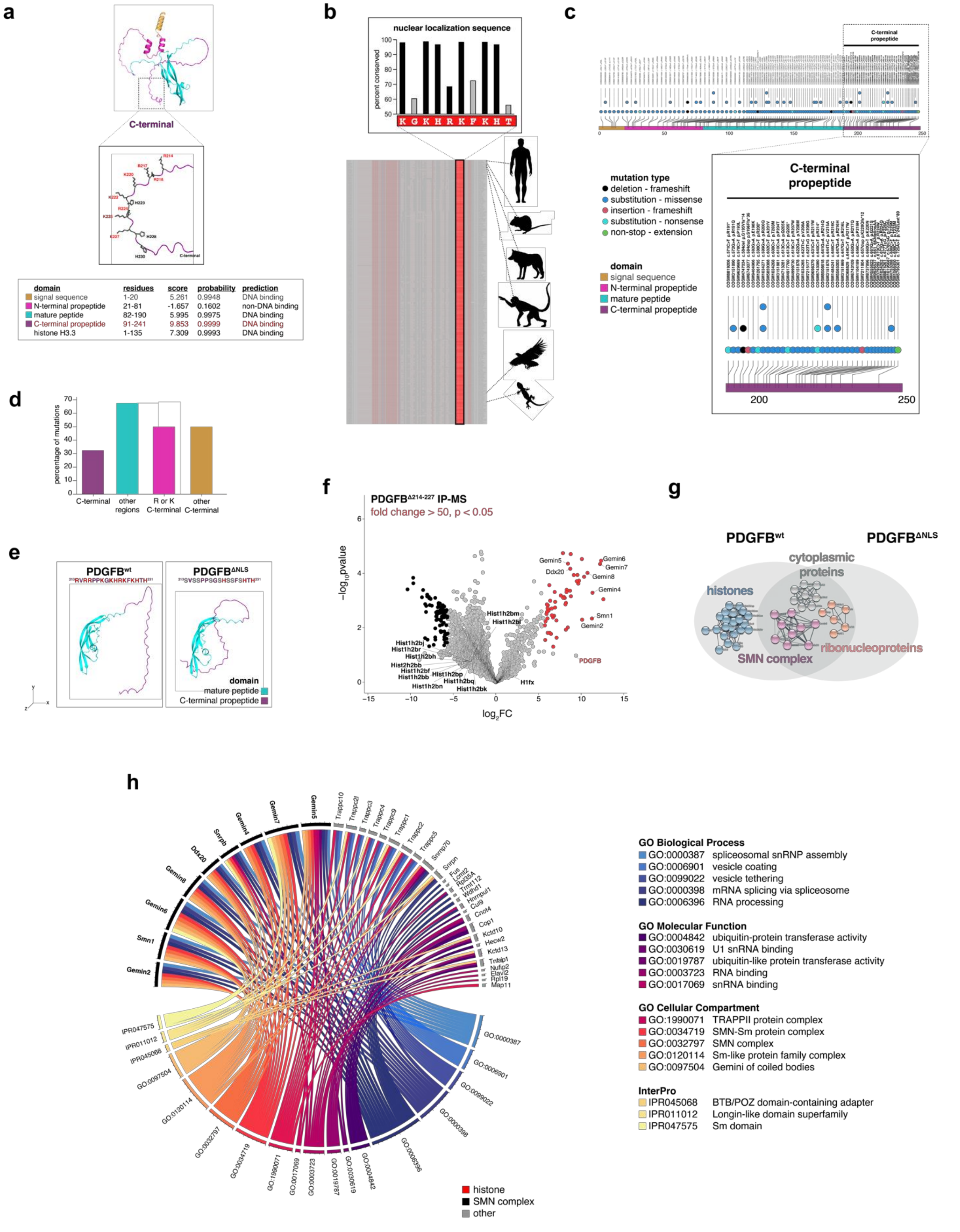

bioRxiv preprint doi: https://doi.org/10.1101/2025.02.20.639365; this version posted February 25, 2025. The copyright holder for this preprint (which was not certified by peer review) is the author/funder, who has granted bioRxiv a license to display the preprint in perpetuity. It is made available under aCC-BY 4.0 International license.

Figure S2. PDGFB contains a highly conserved nuclear localization sequence at its C–terminus. (a) AlphaFold2 modeling of pre-PDGFB. Inset showing basic amino acid residues at the C-terminus and Schematic representation of PDGFB protein domains showing basic amino acid residue enrichment in at the C-terminus. Underlined sequence denotes NLS. Putative DNA-binding prediction scores are shown by protein domain. Histone H3.3 score is shown for reference. (b) Snapshot of PDGFB ortholog alignment across 600 vertebrate genomes shows NLS sequence is highly conserved at most K and R residues. (c–d) Lollipop plot and quantification of TCGA pan-cancer datasets showing the highest incidence of mutations (number of mutations/number of amino acids in each protein domain) is found in the C-terminal domain of PDGFB. (e) AlphaFold2 modeling of the C-terminal containing forms of $PDGFB^{wt}$ and $PDGFB^{\Delta NLS}$ showing conformational changes to the disordered C-terminus upon neutralizing K and R residues. (f) Volcano plot of the 59 proteins identified from PDGFB IP-MS of $PDGFB^{\Delta NLS}$ tumors. Red dots denote proteins with fold change>50; $p<0.05$; black dots denote proteins enriched in IgG fold change<50; $p<0.05$. (g) STRING network of significant proteins from *(f)* showing histone enrichment is no longer detected and schematic representation of significant proteins found in $PDGFB^{wt}$ and $PDGFB^{\Delta NLS}$ tumor brains shows histone binding is only detected in $PDGFB^{wt}$ tumors. (h) Circos plot of GOs ($p<0.05$) corresponding to significant proteins from *(f).* NLS: nuclear localization sequence.

bioRxiv preprint doi: https://doi.org/10.1101/2025.02.20.639365; this version posted February 25, 2025. The copyright holder for this preprint (which was not certified by peer review) is the author/funder, who has granted bioRxiv a license to display the preprint in perpetuity. It is made available under aCC-BY 4.0 International license.

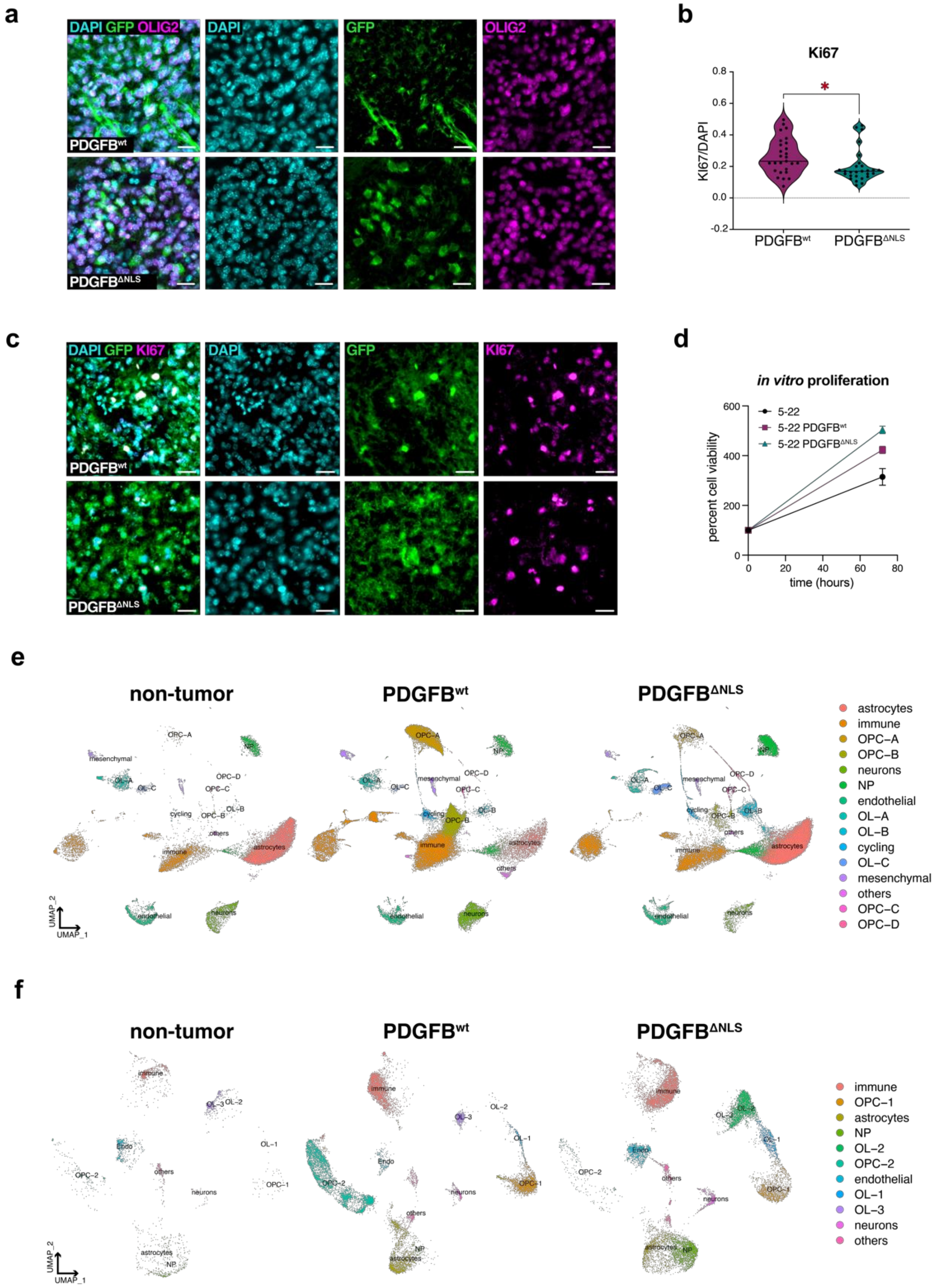

bioRxiv preprint doi: https://doi.org/10.1101/2025.02.20.639365; this version posted February 25, 2025. The copyright holder for this preprint (which was not certified by peer review) is the author/funder, who has granted bioRxiv a license to display the preprint in perpetuity. It is made available under aCC-BY 4.0 International license.

Figure S3. PDGFB drives expansion of OPCs and nuclear PDGFB is required to prevent OL differentiation. (a) Immunostaining of $PDGFB^{wt}$ and $PDGFB^{\Delta NLS}$ tumors shows tumor cells are Olig2+; scale bar = 20 microns. (b) Quantification of Ki67-positivity shows both $PDGFB^{wt}$ and $PDGFB^{\Delta NLS}$ tumors are highly proliferative, with $PDGFB^{wt}$ tumors showing a higher rate of proliferation. (c) Ki67 immunostaining of $PDGFB^{wt}$ and $PDGFB^{\Delta NLS}$ tumors; scale bar = 20 microns. (d) *In vitro* viability assay of mIDH GSCs (5-22) overexpressing $PDGFB^{wt}$ and $PDGFB^{\Delta NLS}$ show increased proliferation as compared to controls. (e) Dim plots of 98,242 cells from our scRNA-seq dataset from $PDGFB^{wt}$ (n=3), $PDGFB^{\Delta NLS}$ (n=3) and non-tumor (n=3) mouse brains showing cell type annotated clusters. (f) Dim plot of 27,836 cells from our scATAC-seq dataset from $PDGFB^{wt}$ (n=1), $PDGFB^{\Delta NLS}$ (n=1) and non-tumor (n=1) mouse brains showing cell type annotated clusters. GSC: glioma stem cell.

bioRxiv preprint doi: https://doi.org/10.1101/2025.02.20.639365; this version posted February 25, 2025. The copyright holder for this preprint (which was not certified by peer review) is the author/funder, who has granted bioRxiv a license to display the preprint in perpetuity. It is made available under aCC-BY 4.0 International license.

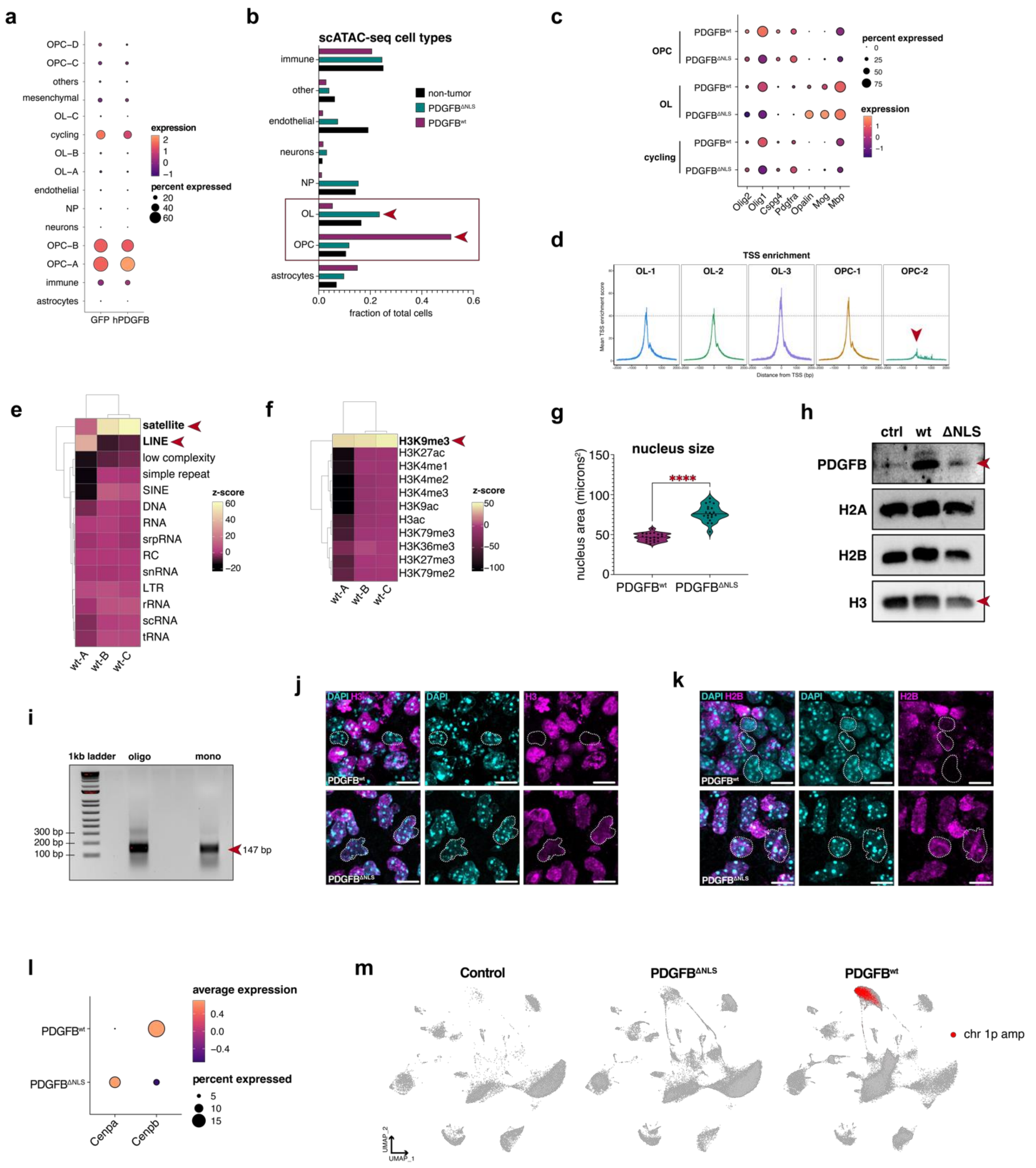

bioRxiv preprint doi: https://doi.org/10.1101/2025.02.20.639365; this version posted February 25, 2025. The copyright holder for this preprint (which was not certified by peer review) is the author/funder, who has granted bioRxiv a license to display the preprint in perpetuity. It is made available under aCC-BY 4.0 International license.

Figure S4. Nuclear PDGFB regulates OPC lineage and heterochromatin architecture. (a) Dot plot of scRNA-seq dataset from PDGFB^{wt}, PDGFB$^{\Delta NLS}$ and non-tumor mouse brains shows OPCs and cycling tumor cells express *GFP* and *PDGFB*. (b) Bar plot of OPC and OL lineage clusters from scATAC-seq showing the OPCs are expanded in PDGFB^{wt} tumors whereas OLs are increased in PDGFB$^{\Delta NLS}$ tumors. (c) Dot plot of OPC, OL and cycling tumor cells from scRNA-seq dataset shows OPCs are enriched for OPC lineage markers in PDGFB^{wt} tumors and OLs are enriched for OL lineage markers in PDGFB$^{\Delta NLS}$ tumors. (d) TSS analysis from scATAC-seq dataset shows OPC-2 cells, which are predominantly found in PDGFB^{wt} tumors, show a large reduction chromatin accessibility. (e–f) Enrichment analysis of PDGFB ChIP-seq from mouse PDGFB^{wt} tumors shows enrichment in PDGFB binding to *(e)* satellite DNA regions (e.g. centromeric repeats), long interspersed nuclear elements (LINEs) and *(f)* H3K9me. (g) Quanitification of nucleus size shows PDGFB^{wt} nuclei are smaller than PDGFB$^{\Delta NLS}$ nuclei. (h) Immunoblot of nucleosome extractions shows PDGFB is detected in the nucleosome fraction and reduced H3 levels in both PDGFB^{wt}- and PDGFB$^{\Delta NLS}$-overexpression wtIDH GSCs (7-2). (i) Image of agarose gel showing single nucleosomes were obtains for the nucleosome extraction immunoblot performed in *(h)*. (j–k) Representative images from *(j)* H3 and *(k)* H2B immunostaining from PDGFB^{wt} and PDGFB$^{\Delta NLS}$ tumors. White dashed lines show select cells with reduced H3 and H2B positivity in PDGFB^{wt} tumors and nuclei with aberrant morphology in PDGFB$^{\Delta NLS}$ tumors. (l) Dot plot from PDGFB^{wt} and PDGFB$^{\Delta NLS}$ mouse tumor scRNA-seq dataset shows *Cenpb* expression is increased in PDGFB^{wt} tumor cells and is reduced when PDGFB cannot enter the nucleus. (m) Feature plots showing scRNA-seq inferred chromosome 1p amplifications are present in PDGFB^{wt} tumors.

bioRxiv preprint doi: https://doi.org/10.1101/2025.02.20.639365; this version posted February 25, 2025. The copyright holder for this preprint (which was not certified by peer review) is the author/funder, who has granted bioRxiv a license to display the preprint in perpetuity. It is made available under aCC-BY 4.0 International license.

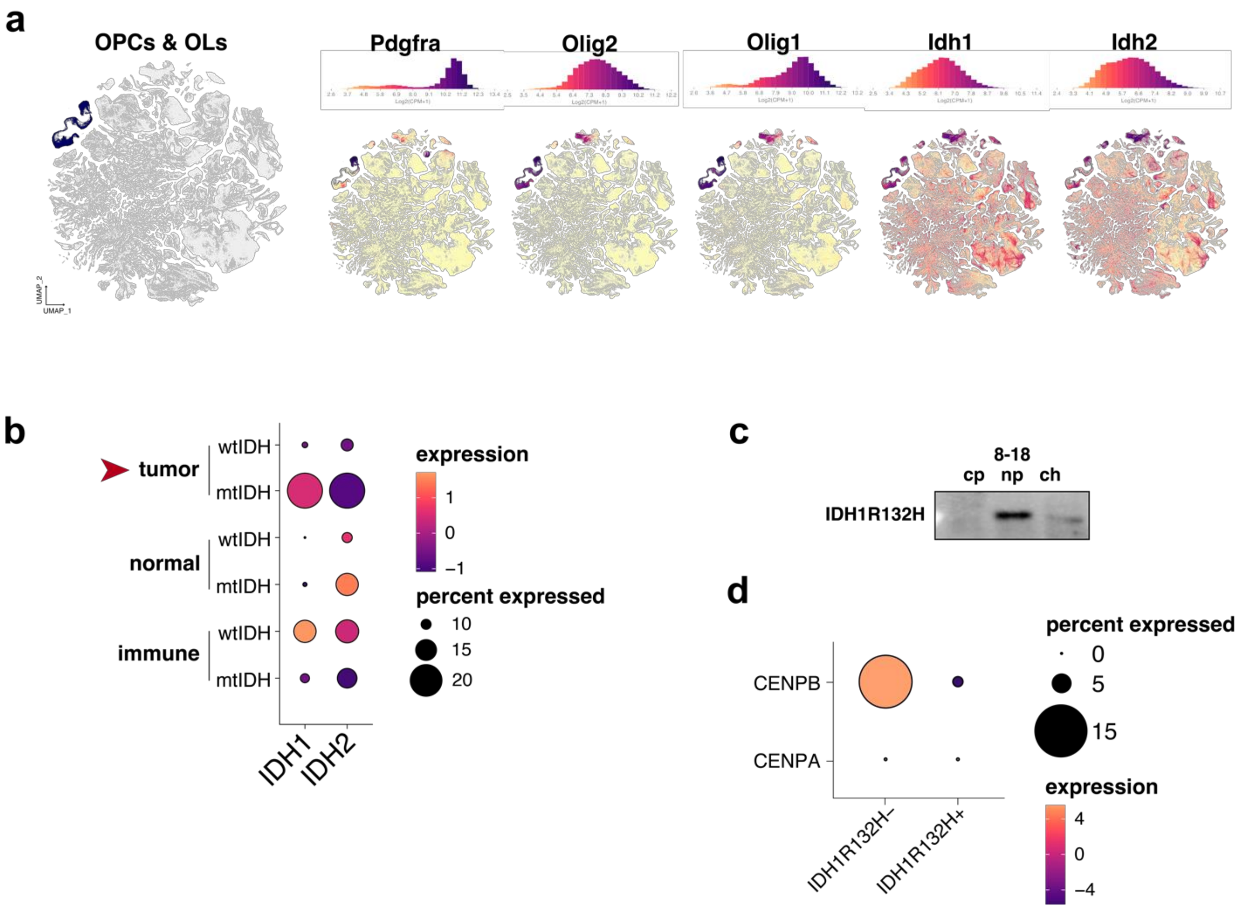

bioRxiv preprint doi: https://doi.org/10.1101/2025.02.20.639365; this version posted February 25, 2025. The copyright holder for this preprint (which was not certified by peer review) is the author/funder, who has granted bioRxiv a license to display the preprint in perpetuity. It is made available under aCC-BY 4.0 International license.

Figure S5. OPCs and mIDH glioma cells are enriched for *IDH1* and *IDH2*. (a) Dim plot and corresponding feature plots are shown for 4.04 million cells from Allen Brain Cell Atlas. Feature plots for OPC and OL lineage markers show *Idh1* and *Idh2* are highly expressed in OPC and OL cells in normal mouse brain. (b) Dot plot of human brain scRNA-seq data from Allen Brain Atlas shows *IDH1* expression is highest in OPCs. (c) Immunoblot of cell fractions from mIDH GSCs (8-18) shows endogenous $IDH1^{R132H}$ is detected in the nucleoplasm. (d) Dot plot from human glioma scRNA-seq dataset showing $IDH1^{R132H}$– cells have increased *CENPB* expression as compared to $IDH1^{R132H}$+ cells.

bioRxiv preprint doi: https://doi.org/10.1101/2025.02.20.639365; this version posted February 25, 2025. The copyright holder for this preprint (which was not certified by peer review) is the author/funder, who has granted bioRxiv a license to display the preprint in perpetuity. It is made available under aCC-BY 4.0 International license.

Table S1 Characteristics of human glioma samples used for this study.

Patient	Tumor Type	IDH Status	1p19q codeletion	WHO Grade	Recurrence	Experiments
WT-01	astrocytoma (GBM)	wild-type	no	IV	primary	ChIP
WT-02	astrocytoma (GBM)	wild-type	no	IV	primary	ChIP
WT-03	astrocytoma (GBM)	wild-type	no	IV	primary	ChIP
WT-04	astrocytoma (GBM)	wild-type	no	IV	primary	ChIP
WT-05	astrocytoma (GBM)	wild-type	no	IV	primary	ChIP
A-01	astrocytoma	mutant	no	II	primary	ChIP
O-01	oligodendroglioma	mutant	yes	II	primary	ChIP
O-02	oligodendroglioma	mutant	yes	II	recurrent	ChIP; ChIP-seq
A-02	astrocytoma	mutant	no	IV	primary	ChIP; ChIP-seq
O-03	oligodendroglioma	mutant	yes	II	primary	ChIP; ChIP-seq
O-04	oligodendroglioma	mutant	yes	III	primary	ChIP
A-03	astrocytoma	mutant	no	III	primary	ChIP; IHC
A-04	astrocytoma	mutant	no	II	primary	IHC
A-05	astrocytoma	mutant	no	IV	primary	IHC
